# The Roles of Type I Interferon in Co-infections With Parasites and Viruses, Bacteria, or Other Parasites

**DOI:** 10.3389/fimmu.2020.01805

**Published:** 2020-10-26

**Authors:** Yuanlin Ma, Xin-zhuan Su, Fangli Lu

**Affiliations:** ^1^Zhongshan School of Medicine, Sun Yat-sen University, Guangzhou, China; ^2^Laboratory of Malaria and Vector Research, National Institute of Allergy and Infectious Disease, National Institutes of Health, Bethesda, MD, United States; ^3^Department of Parasitology, Zhongshan School of Medicine, Guangzhou, China; ^4^Key Laboratory of Tropical Disease Control of Ministry of Education, Sun Yat-sen University, Guangzhou, China

**Keywords:** type I interferon, parasite, virus, bactirum, co-infection

## Abstract

Parasites, bacteria, and viruses pose serious threats to public health. Many parasite infections, including infections of protozoa and helminths, can inhibit inflammatory responses and impact disease outcomes caused by viral, bacterial, or other parasitic infections. Type I interferon (IFN-I) has been recognized as an essential immune effector in the host defense against various pathogens. In addition, IFN-I responses induced by co-infections with different pathogens may vary according to the host genetic background, immune status, and pathogen burden. However, there is only limited information on the roles of IFN-I in co-infections with parasites and viruses, bacteria, or other parasites. This review summarizes some recent findings on the roles of IFN-I in co-infections with parasites, including *Leishmania* spp., *Plasmodium* spp., *Eimeria maxima, Heligmosomoides polygyrus, Brugia malayi*, or *Schistosoma mansoni*, and viruses or bacteria and co-infections with different parasites (such as co-infection with *Neospora caninum* and *Toxoplasma gondii*, and co-infection with *Plasmodium* spp. and *H. polygyrus*). The potential mechanisms of host responses associated with co-infections, which may provide targets for immune intervention and therapies of the co-infections, are also discussed.

## Introduction

Interferons (IFNs) were initially discovered as soluble effector molecules influencing viral replication in 1957 (1). There are three types of IFNs, including type I interferon (IFN-I), type II interferon (IFN-II), and type III interferon (IFN-III). There are 20 subtypes of IFN-I, including 14 subtypes of IFNα, IFNβ, IFNε, IFNκ, IFNτ, IFNδ, and IFNζ, with IFNα and IFNβ being expressed at the highest levels generally (2). Some researchers suggested that IFN-I should contain IFNω, because IFNω shares 62% and 33% amino acid sequence homology with IFNα and IFNβ, respectively (3). All the IFN-I subtypes exert biological activity through common receptors, IFNα/β receptor (IFNAR1 and IFNAR2). IFN-II contains only one subtype, IFNγ, which exerts its biological activity through IFNγ receptor (IFNGR). IFN-III includes four subtypes of IFNλ, e.g., IFNλ1 or named interleukin (IL)-29, IFNλ2 or named IL-28A, IFNλ3 or named IL-28B, and IFNλ4 (4). All the IFN-III subtypes have similar functions to those of IFN-I in antiviral infections and mucosal immunity through IFNλ receptor (IFNLR) (5).

IFN-I binds to IFNARs and activates Janus kinase 1 (JAK1) and tyrosine kinase 2 (TYK2), leading to phosphorylation and subsequent heterodimerization of signal transducers and activators of transcription 1 (STAT1) and STAT2 that bind to IFN regulatory factor 9 (IRF9) to form a complex named IFN-stimulated gene factor 3 (ISGF3) (6). ISGF3 translocates into the nucleus and binds to the IFN-stimulated response element that resides within the promotor region of IFN-stimulated genes (ISGs) (7). It was previously thought that IFN-I and IFN-III had important roles for antiviral infections and mucosal immunity, and IFN-II were mainly involved in antiparasitic infections (5). However, it has been found that IFN-I is also indispensable in anti-parasitic infections (8). In this review, we summarized current understanding on the roles of IFN-I in co-infections with parasites and viruses, bacteria, or other parasites through the searching of PubMed. Our results show that a host's immune response to infection with different parasites can influence susceptibility to infection of other pathogens, and IFN-I play critical roles in host response to co-infections. Elucidation of the molecular mechanisms of IFN-I responses in co-infections will be essential for developing effective therapies for infectious diseases caused by the pathogens.

## The Role of IFN-I in Co-infections With Protozoa and Viruses

### The Roles of IFN-I in Co-infections With *Leishmania* Spp. and Viruses

*Leishmania braziliensis* and *Leishmania guyanensis* often carry a single-segmented double-stranded RNA (dsRNA) *Totivirus* known as *Leishmania* RNA virus 1 (LRV1) (9). In human, LRV1 multiplying within *L. guyanensis* and *L. braziliensis* parasites can cause more severe diseases, more frequent relapse after drug treatment, or increased risk of treatment failure than infections with the parasites alone (10, 11). Co-infection with the lymphocytic choriomeningitis virus (LCMV) or Toscana virus can exacerbate the outcome of *L. guyanensis*-induced murine leishmaniasis and benefit parasite persistence and dissemination (11). Furthermore, LCMV co-infection after healing of leishmaniasis may induce reactivation of disease pathology, overriding the protective adaptive immune response. Mice infected with LRV1-bearing *L. guyanensis* have larger lesions and increased parasite load, and induce a significant down-regulation of IFNGR expression than mice infected with LRV1-cured *L. guyanensis* (11). IFNα and IFNβ have a dose-dependent effect on increasing the lesion size and parasite number in C57BL/6 wild-type (WT) mice infected with *L. guyanensis*. In contrast, IFN-I receptor deficient (IFNAR^−/−^) mice infected with LRV1-bearing *L. guyanensis* have significantly smaller lesions and decreased parasite numbers, similar to those observed in IFNAR^−/−^ and WT mice infected with LRV1-cured *L. guyanensis* (11). Another study tested the effects of co-infection with two *Phlebovirus* isolates, Icoaraci and Pacui from the Amazon region. Increased inflammatory infiltrates with higher IFNβ-expressing cell numbers and parasite burden, and larger lesion size were observed in *Phlebovirus* Icoaraci-*Leishmania amazonensis* co-infected mice than those singly infected with *L*. *amazonensis*. These observations were partially corroborated with *Phlebovirus* Pacui co-infection. *Phlebovirus* Icoaraci aggravates *in vivo L. amazonensis* infection via the engagement of the RNA sensor protein kinase R (PKR) and the expression of IFNβ and IL-10. Furthermore, co-infection with *Phlebovirus* potentiates *in vitro L. amazonensis* infection, and increased macrophage infection requires IFNβ expression induced by the parasite and amplified during co-infection (12). However, dengue virus type-2 co-infection can negatively modulate the intracellular growth of *L. amazonensis* and prevent the expression of IFNβ and IL-10 in macrophages when compared to macrophages infected with *L. amazonensis* only (12). Dengue virus type-2 might impair IFN-I signaling cascade in vertebrate cells leading to defective IFN expression (13, 14). Therefore, viral co-infection can modulate host responses to *Leishmania* infections leading to different disease outcomes, depending on the virus species.

### The Roles of IFN-I in Co-infections With *Plasmodium* Spp. and Viruses

An estimated 228 million cases of malaria occurred worldwide and 405,000 deaths from malaria globally in 2018 (15). It has been reported that *Plasmodium* and respiratory syncytial virus infections remain common among children in sub-Saharan Africa, and that febrile young children concurrently infected with *Plasmodium* and respiratory viral pathogens are less likely to suffer from pneumonia than non-*Plasmodium*-infected children (16). After C57BL/6J mice were simultaneously infected with pneumovirus of mice (PVM) and blood-stage of *Plasmodium chabaudi chabaudi* AS parasites, *P. chabaudi chabaudi* AS infection was unaffected by co-infection with PVM, while PVM-induced weight loss, diminished pulmonary cytokine responses and immune cell recruitment to the airways, and greater viral dissemination throughout the lung were observed by co-infection with *P. chabaudi chabaudi* AS. As a result, *Plasmodium* co-infection induced immunosuppression in the lung is associated with disruption of early systemic IFNβ response to PVM, leading to exacerbating viral dissemination in the lung, which does not occur during infection with PVM alone (17). These data provide evidence that co-infection with *P. chabaudi chabaudi* AS and PVM drives a unique IFNβ response that does not occur during infection with PVM alone. In C57BL/6 mice, it was found that both live blood cells and extracts of blood cells parasitized by *Plasmodium berghei* K173 or *Plasmodium yoelii* 17X YM can protect against *P. berghei* ANKA-induced experimental cerebral malaria (ECM) (18). *P. berghei* K173 triggered an early production of IFNα, CCL4, CCL5, tumor necrosis factor alpha (TNFα), IL-6, and IL-12p40, and the protection was associated with a strong IFN-I signature (18). *P. berghei* K173 and *P. yoelii* 17X YM contain lactate dehydrogenase-elevating virus (LDV), a nonpathogenic mouse virus, which alone protects mice from ECM (18). In ECM, LDV induces a massive IFN-I response, resulting in an IFN-mediated reduction in the number of splenic conventional dendritic cells (cDCs) and an impairment of their ability to produce IL-12p70, leading to a decrease in pathogenic Th1 CD4^+^ T cell responses. *P. berghei* ANKA induced upregulation of the IL-12p35 and IL-12p40 genes, which was blocked by LDV co-infection but rescued in IFNAR1^−/−^ mice. IFNα, IFNβ, TNFα, and IL-6 genes were induced, and splenic T cells and DCs were activated in ECM, which were also reversed in IFNAR1^−/−^ mice (18). Thus, IFN-I are powerful signals that can modify the functionality of many cell types and contribute to the control of *Plasmodium* spp. and viruses co-infection.

### The Roles of IFN-I in Co-infections With *Toxoplasma gondii* and Viruses

*T. gondii* is an obligate intracellular parasite that has a wide intermediate host range including humans (19). Immunocompromised patients infected with HIV are likely to suffer from *T. gondii* infection. To examine how HIV-infected cells were recognized by plasmacytoid dendritic cells (pDCs) and other cells, Lepelley et al. (20) used inhibitors to silence toll-like receptor 7 (TLR7) signaling and found that recognition of virus molecules occurred in primary pDCs and pDC-like cells through TLR7. TTAGGG motifs containing inhibitory oligodeoxynucleotide efficiently block the TLR9 signaling and herpes simplex virus (HSV)-induced IFN-I production by pDCs (21). After recognition, an intracellular signaling pathway is activated in pDCs, including endosomal localization of TLR9 leading to phosphorylation and nuclear translocation of IRF7. The transcription factor IRF7 is essential for the induction of IFNα/β genes via the virus-activated, MyD88-independent pathway, and the TLR-activated, MyD88-dependent pathway (22). *T. gondii* infected human pDCs could functionally inhibit pDCs from producing IFNα responding to HIV-1 or HSV-1 during co-infection (23). The inhibition of IFNα production in response to HSV-1 infection has a dose-dependent effect on the ratio of *T. gondii* to human peripheral blood mononuclear cell counts, whereas uninfected cells responded normally to HSV-1 stimulation. Similar results were found for IFNα production in response to *T. gondii*-HIV co-infections. *T. gondii* rhoptry protein 16 (ROP16) is responsible for inhibition of IRF7 nuclear translocation and inhibition of IFNα production (23). ROP16 knockout RH strain of *T. gondii* failed to inhibit IRF7 nuclear translocation as compared to the WT RH strain. pDCs from mice infected with ROP16 knockout *T. gondii* produced more IFNα than those infected with WT parasites, indicating that ROP16-mediated phosphorylation of STAT3 is an important mechanism for *T. gondii* inhibition of IFNα production. IL-10 also inhibits IRF7 nuclear translocation in pDCs after stimulation with HSV-1, which is one of the important mechanisms for *T. gondii* inhibition of IFNα production. *T. gondii* suppresses pDC activation by mimicking IL-10's regulatory effects through an ROP16 kinase-dependent mechanism (23). These data provide evidence of inhibition of innate immune responses to HIV-1 and HSV-1 by *T. gondii* through inhibition of IFNα production.

## The Roles of IFN-I in Co-infections With Helminths and Viruses

### The Roles of IFN-I in Co-infection With *Heligmosomoides polygyrus* and Virus

Mice co-infected with the gastrointestinal helminths *Trichinella spiralis* or *H. polygyrus* and mouse norovirus showed increased viral loads and reduced levels of virus-specific CD4^+^ T cells expressing IFNγ and TNFα than mice infected with norovirus alone, indicating helminth infection suppresses antiviral immunity (24). However, *H. polygyrus* infection can also induce protective immunity against viral infections. McFarlane et al. (25) infected mice with 200 3rd stage larvae of *H. polygyrus* by oral gavage and then infected the mice with respiratory syncytial virus (RSV) or UV-inactivated RSV by intranasal dropping 10 days later. It was found that enteric *H. polygyru* infection up-regulated expressions of IFN-I and ISGs in both the duodenum and the lung, resulting in the reduction of RSV load as well as pulmonary inflammation. In germ-free IFNAR1^−/−^ mice, the protection (e.g., reduction of RSV load and the induction of ISG) was lost, indicating *H. polygyrus*-induced protection against RSV infection requires IFNAR signaling. *H. polygyrus* adult excretory and secretory products are not responsible for the effects of RSV infection, while larval stages alone are required to protect against RSV infection (25). Two ISGs, *oas* and viperin, have been found to be driven by IFN-I and play a protective role in RSV infection (26, 27). These data demonstrate that enteric helminth infection can have protective antiviral effects in the lung through induction of RSV-dependent IFN-I response.

### The Roles of IFN-I in Co-infection With *Schistosoma mansoni* and Viruses

*Schistosoma* spp. are responsible for human schistosomiasis. C57BL/6 mice were percutaneously infected with 25 or 50 *S. mansoni* cercariae, and then were intravenously inoculated with 10^5^ PFU of a hepatotropic strain of LCMV (WE2.2) 10 weeks later. Schistosome egg antigens were shown to suppress IFN-I response by DCs and enhance intrahepatic LCMV replication in the co-infected mice (28). On the other hand, the co-infected mice showed a significantly higher hepatic egg burden, leading to a dramatic increase in morbidity and mortality associated with the increased serum levels of aspartate transaminase (AST), alanine transaminase (ALT), and nitric oxide (NO). Compared to mice infected with schistosome or LCMV alone, the high serum AST and ALT levels in the co-infected mice indicate a substantial degree of liver damage, likely due to increased serum NO production in the co-infected mice (28). The results are similar to the observations in a study of 126 Egyptian patients, in which patients co-infected with hepatitis C virus (HCV) and *S. mansoni* have more advanced liver disease, with higher HCV RNA titers, higher hepatic inflammatory and fibrosis/cirrhosis scores, higher incidence of hepatocellular carcinoma, and higher mortality rate than patients with HCV infection alone (29). Furthermore, HCV and *S. mansoni* co-infection had either no or weak CD4^+^ T cell response compared to patients with HCV infection alone (30). Therefore, co-infection of viruses can affect the parasite infection, and vice versa. Pegylated-IFNα-2a (PEG-IFNα) is a standard treatment for HCV infection and can reduce granuloma size and adult parasite counts in *S. mansoni*-mono-infected albino mice. PEG-IFNα treatment results in significantly (35%) lower total worm burdens and up to 80% lower egg count in the livers. Interferon treatment also increases the proportion of single worms over parasites in pairs (31). However, a retrospective analysis of 3,596 chronic HCV patients in Egypt, who received PEG-IFNα after antischistosomal therapy for schistosomiasis, showed that positive schistosomal serology has no effect on fibrosis staging but is significantly associated with failure of response to HCV treatment despite antischistosomal therapy (32). Because schistosomiasis is significantly associated with failure to respond to HCV treatment, *Schistosoma* infections diagnosed by serology should be considered for chronic HCV patients prior to initiating PEG-IFNα therapy.

## The Roles of IFN-I in Co-infections of Protozoa With Bacteria

### The Roles of IFN-I in Co-infection With *Eimeria maxima* and Bacteria

*Clostridium perfringens* is a Gram-positive (G^+^) pathogenic bacterium and the causative agent of necrotic enteritis (33). Co-infection with *C. perfringens* and *E. maxima*, a protozoan parasite living in the gut and causing coccidiosis in poultry, has been strongly implicated in promoting necrotic enteritis (34). In one study, 3-week-old chickens were orally infected with 5 × 10^4^ sporulated oocysts of *E. maxima*, and then orally infected with 10^9^ *C. perfringens* bacteria 5 days later. The results showed a synergistic relationship during the course of experimental necrotic enteritis. *E. maxima*/*C. perfringens* co-infection resulted in more severe intestinal pathology, reduced body weight gain, increased numbers of intestinal *C. perfringens* bacteria, and altered cytokine/chemokine expression compared to chickens exposed to *E. maxima* or *C. perfringens* alone (35). Co-infection of *E. maxima* with *C. perfringens* suppressed the expression of IFNα, IFNγ, IL-1β, IL-2, IL-12, IL-13, IL-17, NO, and transforming growth factor-β4 genes, but increased the expression of IL-8, IL-10, IL-15, and lipopolysaccharide-induced TNFα factor (35). Therefore, IFNα is considered to have a role in disease severity upon co-infection of *E. maxima* with *C. perfringens*.

### The Roles of IFN-I in Co-infection With *P. falciparum* and Bacteria

Co-infections with bacteria and malaria parasites are common life-threatening conditions in children residing in sub-Saharan Africa (36). It has been reported that the most frequently isolated G^+^ and Gram-negative (G^−^) bacteria found from 206 children aged 3–36 months in Kenya are *Staphylococcus aureus* (G^+^ bacteria) and non-Typhi *Salmonella* (G^−^ bacteria), respectively (36). Kenyan children co-infected with G^−^ bacteria and *P. falciparum* can increase clinical outcomes such as malnutrition, respiratory distress, anemia, and mortality, although bacteremia is associated with reducing incidences of high-density parasitemia (37). Severe anemia in malaria patients is not affected by co-infection with G^−^ bacteria, and *P. falciparum* burden in G^−^ bacteria co-infected patients has five-fold lower median parasitemia and six-fold lower geometric mean parasitemia than those of *P. falciparum*-mono-infected patients. In addition, cytokines including IL-4, IL-5, IL-7, IL-12, IL-15, IL-17, IFNα, and IFNγ are higher in patients co-infected with *P. falciparum* and G^−^ or G^+^ bacteria than *P. falciparum* mono-infected patients. Parasitemia is inversely associated with elevated levels of IL-1β, IL-1Ra, IL-12, IL-15, IL-17, IFNα, and IFNγ in children co-infected with *P. falciparum* and G^−^ bacteria, suggesting that the cytokines may induce anti-parasitic activities (36). Furthermore, children with *P. falciparum*-mono-infection had significantly depressed levels of IFNα compared to children with G^−^ or G^+^ bacteria and *P. falciparum* co-infections (36). These data suggest that enhanced immune activation, especially increased serum level of IFNγ and IFNα in co-infected children, may act to reduce malaria parasite densities in these individuals.

## The Roles of IFN-I in Co-infections With Helminth and Bacteria

### The Roles of IFN-I in Co-infection With *Brugia malayi* and Bacteria

*Brugia malayi* is a parasitic nematode and etiological agent of lymphatic filariasis. Human DCs infected with *Mycobacterium tuberculosis* produce IL-1β, IL-12, IL-18, IFNα, and TNFα to activate T cells and trigger adaptive immunity (38). Similarly, human macrophages infected with *M. tuberculosis* produce IL-1β, IL-6, IL-10, IL-18, and TNFα (38). Activated macrophages can kill *M. tuberculosis* in the presence of IFNγ produced by activated T cells (39). In one study, human DCs and macrophages were exposed *in vitro* to 12,500 live *B. malayi* microfilariae on day 7 and then were infected with *M. tuberculosis* strain H37Rv on day 9. It was found that pre-exposure of *B. malayi* microfilariae decreased the expression of IL-10, IFNα, and macrophage inflammatory protein-1β by DCs and the expression of IL-10 and IFNα by macrophages after infection with *M. tuberculosis* (40). IL-10 and IFNα are important for treating tuberculosis, and the decreased expression of IL-10 and IFNα can lead to a significant impairment of antimicrobial activity (41). When cultured with autologous CD4^+^ T cells, live microfilariae-exposed and *M. tuberculosis*-infected DCs were less capable of stimulating IFNγ production, leading to a significant impairment in limiting mycobacterial growth (40). These data demonstrate that filarial parasites can affect the functions of DCs and macrophages and have an impact on the outcome of concurrent *M. tuberculosis* infection.

## The Role of IFN-I in Co-infection With Different Protozoa

### The Roles of IFN-I in Co-infection With *Neospora caninum* and *T. gondii*

*N. caninum* is an intracellular protozoan that infects many animals, and is similar to *T. gondii* in morphology and developmental stages, but there are differences in many genes between these two parasites (42). In an *in vitro* study, after pre-infection with *N. caninum*, human fibroblasts can induce a strong IFN-I response to control *T. gondii* infection, but not vice versa. In contrast, pre-infection with *T. gondii* for 1 h followed by heat-killed *N. caninum* failed to induce human fibroblasts to produce IFN-I (43). Therefore, IFN-I induced by atypical strains of *T. gondii* and/or *N. caninum* can cause suppression of IFNγ production and more severe disease (8). The results demonstrate that *N. caninum* is a potent inducer of innate IFNα/β responses, and that *T. gondii* has the capacity to suppress the responses.

### The Roles of IFN-I in Co-infection With Protozoa and Helminth

To investigate whether *Plasmodium* infections compromise anti-helminth immunity, Coomes et al. (44) infected C57BL/6 mice with 200 3rd stage larvae of *H. polygyrus* by oral gavage and then intraperitoneally injected 10^5^ *P. chabaudi chabaudi* AS parasitized red blood cells into the mice 6 days later. Reduced anti-helminth Th2 cell responses and compromised anti-helminth immunity were found during *H. polygyrus* and *P. chabaudi* co-infections, which protected recipient mice from high parasitemia of malaria parasites. Both IFN-I responsive and unresponsive Th2 cells can up-regulate IFNγ and increase serum IFNγ levels, providing protection against high parasitemia similarly observed in either IFNAR^−/−^ or IFNAR^+/+^ mice (44). However, in another study, IFN-I was shown to be essential for IFNγ production from converted Th2 cells (45). The study indicates that *Plasmodium* infection can negatively affect anti-helminth responses by reducing Th2 cell reaction.

## Molecular Signaling for IFN-I Response to Co-infections

### IFN-I Response to Co-infection With Parasites and Viruses

Recognition of pathogen-associated molecular patterns by pathogen recognition receptors (PRRs) such as TLRs leads to production of IFN-I. TLRs are some of the most important PRRs, and pDCs are one of the major cells producing IFN-I (23). In *P. chabaudi chabaudi* AS and PVM co-infected mice, activation of pDCs was boosted via several PRRs such as TLR7 in pDC recognition of PVM, and TLR9 and stimulator of IFN genes (STING) through recognition of AT-rich DNA bound to *Plasmodium*-derived hemozoin (46–48). In *P. berghei* ANKA and LDV co-infected mice, LDV nucleic acid was recognized by TLR7 and induced a rapid systemic IFN-I production by pDC (49). IFN-I signaling crippled cDC quantitatively and qualitatively, leading to a decrease of IL-23 production that is responsible for the pathogenic polarization of CD4^+^ T cells in *P. berghei* ANKA-induced ECM (18). Genes known to be stimulated by IFN-I, including those encoding 2′-5′-oligoadenylate synthetase (*oas1a*), *oas2, oasl2*, or *pkr*, are significantly up-regulated in mice co-infected with LRV1-bearing *L. guyanensis* or LRV1-cured *L. guyanensis* and LCMV, compared to mice infected with LRV1-cured *L. guyanensis* alone (11). The dsRNA of LRV1 acts to promote *L. guyanensis* virulence through TLR3 and IFN-I signaling (11, 50). *L. guyanensis* parasites with a high LRV1 burden promote TLR3-dependent secretion of proinflammatory cytokines, chemokines, and IFNβ (51). Although miRNA-155 is the only microRNA up-regulated in macrophages in the presence of LRV1 in a TLR-3/TIR-domain-containing adaptor-inducing IFNβ (TRIF)-dependent manner, the secretion of pro-inflammatory cytokines (TNFα, IL-6, and IFNβ) and the mRNA expression of IFNβ or the phosphorylation of IRF-3 of primary murine macrophages are not dependent on miRNA-155 in response to poly I:C or co-infection with *L. guyanensis* and LRV1 (50). *Phlebovirus* Icoaraci reduced PKR phosphorylation that induced by *L. amazonensis* at 1 and 4 h post infection. Furthermore, *Phlebovirus* Icoaraci induced and sustained PKR activation, leading to enhanced IL-10 expression, suggesting a synergism between *L. amazonensis* and *Phlebovirus* Icoaraci (12). The innate IFNα response induced via TLR7/9 by HIV-1 or HSV-1 in human pDCs is suppressed by ROP16 kinase of *T. gondii* through IL-10 mediated inhibition of HSV-induced IRF7 nuclear translocation (23). In *H. polygyrus* and RSV co-infected mice, *H. polygyrus* infection induces up-regulation of IFNβ transcription and IFNα protein levels in the lung at very early time points after infection. Using IFNAR1^−/−^ mice, enteric helminth infection has protective antiviral effects in the lung through induction of a microbiota-dependent IFN-I response, which is critical to anti-RSV immunity (25). Alveolar macrophages are the major source of IFN-I upon RSV infection in mice (52), and rapid and strong IFN-I and ISG responses can be induced upon stimulation of a virus such as RSV (53). Schistosome eggs contained dsRNA that is able to trigger DC activation via TLR3 (54). Schistosome eggs induce IFNβ expression and trigger IFNAR expressed on DCs, resulting in phosphorylation of STAT1 and up-regulation of IFN- induced inflammatory products (55). However, in *S. mansoni* and LCMV co-infected mice, schistosome egg antigens suppress IFN-I response in DCs induced by LCMV (28).

### IFN-I Response to Co-infection With Parasites and Bacteria

Malarial parasite DNA bound to hemozoin pigment is targeted to intracellular compartments to activate TLR9 (56), and TLR9 and MyD88 play central roles in the immune regulation and development of protective immunity to malaria (57). DNA from the parasites can be recognized by cytosolic sensors such as cyclic GMP-AMP synthase (cGAS) through STING, TBK1, and IRF3-IRF7 pathway to induce IFN-I that can recruit natural killer T cells to produce IFNγ (8, 48, 58). Mice deficient in cGAS, STING, MDA5, MAVS, or IRF3 produced high amounts of IFN-α/β in the serum and are resistant to lethal *P. yoelii* YM infection, suggesting that activation of cGAS-STING and MDA5-MAVS-mediated IRF3-dependent IFN-I signaling leads to a lethal *P. yoelii* YM infection (58). Furthermore, pDCs, cDCs, and macrophages are required for generating IFN-α/β-induced subsequent protective immunity and suppressor of cytokine signaling 1 works as a key negative regulator to inhibit MyD88-dependent IFN-I signaling in pDCs. These data identify a critical regulatory mechanism between different IFN-I signaling pathways in pDCs as well as a critical role of stage-dependent IFN-I production in developing protective immunity (58). IFN-I were induced in human primary CD14^+^ monocytes purified from peripheral blood mononuclear cells in response to *P. falsiparum* genomic DNA (59). In addition, IFNα and IFNγ are significantly increased in patients co-infected with *P. falciparum* and G^−^ or G^+^ bacteria compared to *P. falciparum* mono-infected patients. IFNα plays an important role in parasite clearance possibly via induction of NO, which is associated with reduced parasitemia (60). Further studies are needed to understand the molecular mechanisms responsible for the role of IFN-I in malaria and bacterial infection.

In co-infection with *B. malayi* and *M. tuberculosis*, the decreased function of DCs and macrophages to migrate and present antigens to T cells results in decreased expression of cytokines such as IL-10, IFNα, and IFNγ (40). In *E. maxima* and *C. perfringens* co-infected chickens, the expressions of IFNα, IFNγ, and NO are repressed, while IL-10 expression is up-regulated (35). IL-10 inhibits IFNγ expression and prevents the development of IFNγ-driven responses, which is crucial for control of *E. maxima* infection (61). *Clostridium orbiscindens* produced desaminotyrosine that primes the amplification loop of IFN-I signaling to protect the host (62). However, whether *C. perfringens* can also produce desaminotyrosine needs to be further studied.

### IFN-I Response to Co-infection With Different Parasites

In *T. gondii* and *N. caninum* co-infected mice, *N. caninum* RNA can induce IFN-I responses to *N. caninum* or heat-killed *T. gondii* via TLR3 and TRIF, suggesting that parasite RNA is a potential trigger for this response (43). TLR3-dependent induction of IFN-I responsive genes can be elicited by the transfection of macrophages with *N. caninum* RNA, but not with *T. gondii* RNA (43). Profilin of *T. gondii* is the ligand for TLR11 and TLR12 and boosts host immune responses through the activation of IL-12 and IFNα in mice (63, 64).

## Concluding Remarks

Co-infections with parasites and viruses, bacteria, or other parasites are quite common in patients. Increasing evidences show that co-infections with these infectious agents may alter host immune responsiveness and disease outcomes. IFN-I plays important roles during the co-infections, which can either deteriorate or attenuate the consequence of diseases via the interactions. However, so far the literatures about IFN-I in co-infections with parasites and viruses, bactera, or other parasites are limited. A better understanding of how the susceptibility and pathogenesis of infectious disease can be influenced by parasite co-infections, and the mechanisms of IFN-I response in co-infection may yield new therapies for more effective control of the infectious diseases.

## Author Contributions

YM wrote the manuscript draft. X-zS revised and edited the manuscript. FL conceived and wrote the manuscript. All authors contributed to the article and approved the submitted version.

## Conflict of Interest

The authors declare that the research was conducted in the absence of any commercial or financial relationships that could be construed as a potential conflict of interest.

## Abbreviations

ALT: alanine transaminase
AST: spartate transaminase
cGAMP: cyclic GMP-AMP
cGAS: cyclic GMP-AMP synthase
DC: dendritic cell
gDNA: genomic DNA
G^−^: Gram-negative
G^+^: Gram-positive
HCV: hepatitis C virus
HIV: human immunodeficiency virus
HSV: herpes simplex virus
IFN: interferon
IFNAR: IFNα/β receptor
IFNAR^−/−^: type I IFN receptor deficient
IFNGR: IFNγ receptor
IFN-I: type I interferon
IFN-II: type II interferon
IFN-III: type III interferon
IFNLR: IFNλ receptor
IL: interleukin
IRF: IFN regulatory factor
ISGF3: IFN-stimulated gene factor 3
ISGs: IFN-stimulated genes
JAK1: Janus kinase 1
LCMV: lymphocytic choriomeningitis virus
LDV: lactate dehydrogenase-elevating virus
LRV1: *Leishmania* RNA virus 1
NO: nitric oxide
*oas*: 2'-5'-Oligoadenylate synthetase
pDC: plasmacytoid dendritic cell
PEG-IFNα: Pegylated-IFNα-2a
*pkr*: protein kinase R
PRR: pathogen recognition receptor
PVM: pneumovirus of mice
ROP16: rhoptry protein 16
RSV: respiratory syncytial virus
STAT: signal transducers and activators of transcription
STING: stimulator of IFN genes
TLR: toll-like receptor
TNFα: tumor necrosis factor α
TRIF: TIR-domain-containing adaptor-inducing IFNβ
TYK2: tyrosine kinase 2
WT: wild-type.
